# Counting Grasping Action Using Force Myography: An Exploratory Study With Healthy Individuals

**DOI:** 10.2196/rehab.6901

**Published:** 2017-05-16

**Authors:** Zhen Gang Xiao, Carlo Menon

**Affiliations:** ^1^ Simon Fraser University Burnaby, BC Canada; ^2^ Schools of Mechatronics Systems Engineering and Engineering Science Simon Fraser University Surrey, BC Canada

**Keywords:** myography, classification, upper extremity, grasp, rehabilitation

## Abstract

**Background:**

Functional arm movements generally require grasping an object. The possibility of detecting and counting the action of grasping is believed to be of importance for individual with motor function deficits of the arm, as it could be an indication of the number of the functional arm movements performed by the individuals during rehabilitation. In this exploratory work, the feasibility of using armbands recording radial displacements of forearm muscles and tendons (ie, force myography, FMG) to estimate hand grasping with healthy individuals was investigated. In contrast to previous studies, this exploratory study investigates the feasibility of (1) detecting grasping when the participants move their arms, which could introduce large artifacts to the point of potentially preventing the practical use of the proposed technology, and (2) counting grasping during arm-reaching tasks.

**Objective:**

The aim of this study was to determine the usefulness of FMG in the detection of functional arm movements. The use of FMG straps placed on the forearm is proposed for counting the number of grasping actions in the presence of arm movements.

**Methods:**

Ten healthy volunteers participated in this study to perform a pick-and-place exercise after providing informed consent. FMG signals were simultaneously collected using 2 FMG straps worn on their wrist and at the midposition of their forearm, respectively. Raw FMG signals and 3 additional FMG features (ie, root mean square, wavelength, and window symmetry) were extracted and fed into a linear discriminant analysis classifier to predict grasping states. The transition from nongrasping to grasping states was detected during the process of counting the number of grasping actions.

**Results:**

The median accuracy for detecting grasping events using FMG recorded from the wrist was 95%, and the corresponding interquartile range (IQR) was 5%. For forearm FMG classification, the median accuracy was 92%, and the corresponding IQR was 3%. The difference between the 2 median accuracies was statistically significant (*P*<.001) when using a paired 2-tailed sign test. The median percentage error for counting grasping events when FMG was recorded from the wrist was 1%, and the corresponding IQR was 2%. The median percentage error for FMG recorded from the forearm was 2%, and the corresponding IQR was also 2%. While the median percentage error for the wrist was lower than that of the forearm, the difference between the 2 was not statistically significant based on a paired 2-tailed sign test (*P*=.29).

**Conclusions:**

This study reports that grasping can reliably be counted using an unobtrusive and simple FMG strap even in the presence of arm movements. Such a result supports the foundation for future research evaluating the feasibility of monitoring hand grasping during unsupervised ADL, leading to further investigations with individuals with motor function deficits of the arm.

## Introduction

Individuals with motor function deficits of their arm (eg, individuals with stroke and a hemiparetic arm) often involuntarily avoid using their weak arm during the activities of daily living (ADL)[1,2], leading to an inevitable and gradual degradation of their ability to move their arm. Published studies have shown that increasing the use of a person’s weak arm is believed to be an important factor for a successful recovery [3]. Technologies that provide objective feedback to the individual on the use of their arm could potentially encourage them to be more proactive in using their affected arm [4], and consequently gradually improve their arm motor functions.

Some studies have used accelerometer-based devices to capture gross movements of the arm [3,5,6] and provide that information as activity feedback to the individual. However, such devices generally cannot discern between movements that are functional and believed to be relevant for the recovery [7,8] (ie, grasping a glass and drinking) from those that are not functional (ie, arm movements induced by movements of the body, such as turning, walking, moving during sleeping) [1,3]. Therefore, accelerometer-based devices could provide inaccurate and potentially counterproductive feedback [1].

Devices with the ability to detect grasping motions, which is generally required during functional arm movements, could potentially provide a more suitable indication of functional use of the upper limb [2,9]. Studies showed that exercising the arm by grasping an object has the potential to greatly improve rehabilitation outcomes [10,11]. Hence, detecting the number of grasping actions performed by an individual during ADL could be used as feedback to facilitate rehabilitation. In addition to uses for rehabilitation, the ability to unobtrusively detect grasping motions could be used in applications such as monitoring the repetitive hand activity level of a worker for load transfer tasks [12], or could be used in identification of hand-held objects [13]. Hence, innovative solutions to detect grasping motions are, therefore, in need.

Currently, commercial devices capable of detecting grasping motions do exist. They are mostly based on either a vision-based approach or using a wearable technology approach such as wearing data gloves. The vision-based systems, such as Microsoft’s Kinect [14], Leap Motion Controller [15], and Optotrak [16], have to be mounted externally to the user’s body and are generally used in well-controlled indoor environments such as a rehabilitation center or clinic. They cannot be used to monitor ADL, especially when the individual is in an outdoor setting. On the other hand, the use of a data glove, such as the CyberGlove [17], can be used for monitoring grasping motions outdoors. However, data gloves are generally not practical for use by individuals with a clutched hand, as in the case of individuals with a hemiparetic arm resulting from stroke. In fact, they require a considerable effort to be donned and doffed. Furthermore, they cannot be used in many ADL, such as washing dishes, taking a shower, etc, as they are not waterproof or are simply uncomfortable. Data gloves also reduce the tactile sensation of the hand and fingers, which poses a further barrier for being accepted by the users [18,19].

In addition to the above-mentioned commercially available technologies, the academic community has investigated different approaches to classify grasping and other hand gestures. One of these approaches is based on surface electromyography (sEMG) recorded from the forearm [20]. While such an approach could potentially be used in a large variety of environments, including outdoors, its signal quality may degrade due to many environmental factors, such as sweating and electrical noise, which has been shown to drastically affect its performance [21]. Furthermore, medical-grade sEMG systems capable of capturing low noise sEMG signals generally cost thousands of US dollars (eg, Noraxon sEMG system), which makes them unsuitable for being implemented in practice.

An alternative approach to detect grasping is force myography (FMG). FMG is a technique that uses sensors to capture displacements of muscles, skin, and tendons [22]. This technique was also referred to as topographic force mapping [21], residual kinetic imaging [23], or muscle pressure distribution mapping [24]. Although the FMG technique is relatively unexplored and less standardized compared with sEMG, it presents different potential advantages over the latter. Specifically, FMG signals do not degrade due to sweating or electrical noise [21]. As a consequence, the related hardware for the signal acquisition is also less sophisticated and expensive. While FMG devices are currently not commercially available, an experimental prototype of an FMG signal acquisition device costs less than US $50 [25].

The use of FMG can be dated back as early as the 1960s, when FMG was proposed for controlling a single-degree prosthetic terminal device [26]. Since then, the use of FMG for controlling hand prosthesis has gained some interest in the research community [27-33]. At the same time, researchers also explored the use of FMG for individuals with intact limbs for various applications. For example, FMG signals taken from healthy individuals were studied for regressing isometric force applied by the fingers [22,25], as well as for recognizing different hand gestures and finger movements [24,31,34,35]. Also, a preliminary test performed by Yungher and Craelius showed that regressing the grasping force through the forearm FMG of individuals with poststroke condition was viable [36]. Recently, robotic orthosis with FMG sensing capability were proposed for potential stroke rehabilitation applications [37,38].

FMG signals to detect hand movements are generally extracted from the middle of the forearm where large radial displacements can be recorded [22,24,25,31,34,39-42]. Recent studies have, however, explored the possibility of estimating hand movements by processing FMG signals recorded at the wrist. For instance, Morganti et al proposed a wrist strap consisting of four force-sensing resistors (FSRs) to detect wrist positions [43]. Dementyev and Paradiso subsequently developed a wrist strap that was capable of deciphering 6 static hand gestures [44]. The works of both Morganti and Dementryev showed the potential of embedding FMG sensors inside a watch strap, which could make the technology acceptable for users, especially those who may highly value the cosmetics of the device.

The large majority of studies on FMG for the upper extremities presented in the literature considered exploratory tests in very controlled scenarios, where healthy volunteers were asked to move their hand or wrist while maintaining a fixed elbow position [21,22,24,25,29,31,35,43-46]. Despite being able to obtain high prediction accuracy, this approach does not truly reflect the capability of FMG for detecting hand action in a practical scenario, in which arm movements are generally present. To the best of the authors’ knowledge, the only studies that included arm movements with the use of FMG are the ones performed by Ogris et al [47] and Sadarangani and Menon [48]. Both of their studies used FMG in conjunction with inertial measurement unit (IMU) to decipher various ADL. The result of Ogris’s work showed that FMG improved classification accuracies of some ADL; however, the capability of using FMG to decipher hand action was not fully investigated. In the other study, Sadarangani and Menon’s work focused on the investigation of detecting hand actions, but only for 3 limited scenarios.

As a fundamental step toward the development of a technology suitable to detecting grasping motions in the presence of arm movements as an indirect estimation of functional arm movements, this study investigates the ability of using FMG to count the number of grasping motions during a series of pick-and-place (PAP) actions. Two wireless FMG straps were prototyped and placed close to the wrist and on the forearm of 10 healthy individuals for this study.

## Methods

### FMG Signal Extraction and Data Transmission

**Figure 1:**
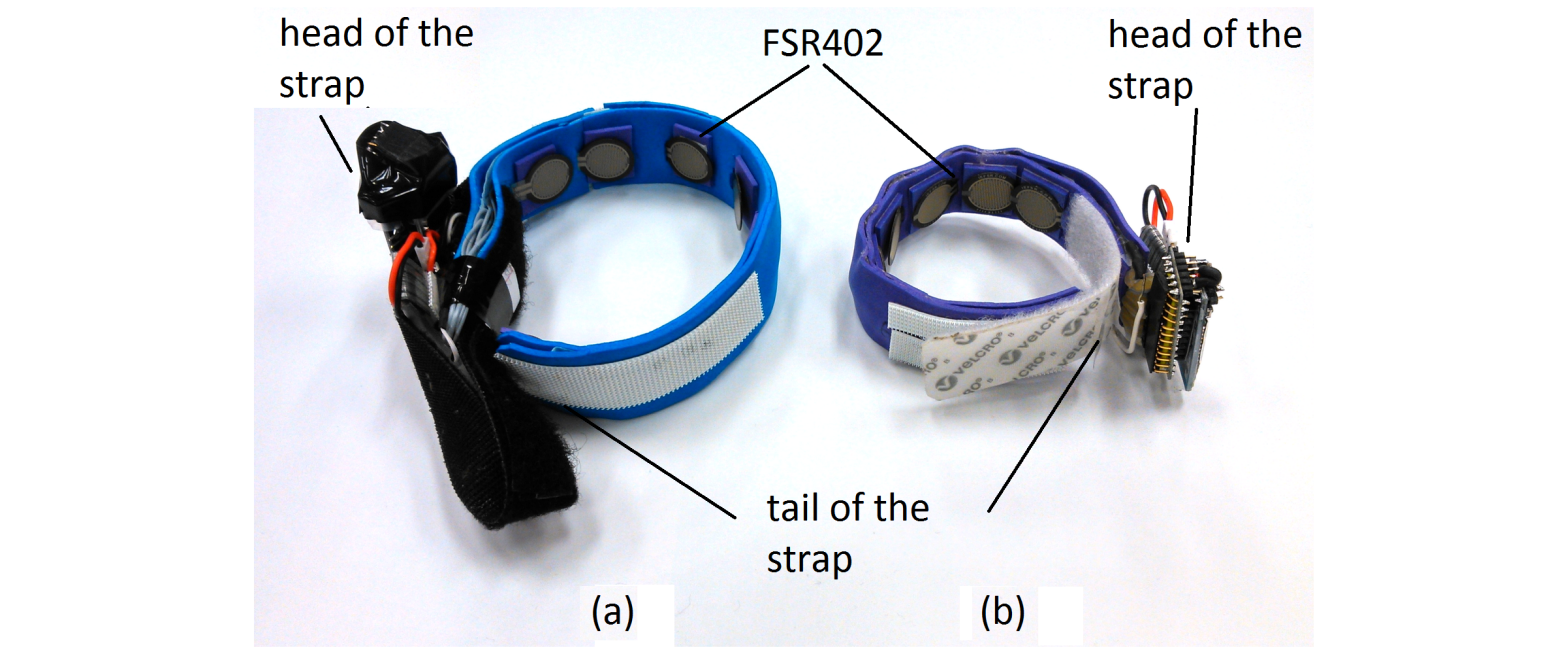
Wireless FSR straps: (a) wireless FSR strap for the forearm and (b) wireless FSR strap for the wrist.

Figure 1 shows the 2 FMG strap prototypes used in this study. The strap in the left of the figure is 28 cm long and it was designed to be donned on the forearm while the strap in the right is shorter (19 cm) and was designed to be donned on the wrist, like a watch. Each strap had 8 FSR sensors (FSR 402 from Interlink Electronics), which were evenly distributed on the straps’ inner surface (see Figure 1).

**Figure 2:**
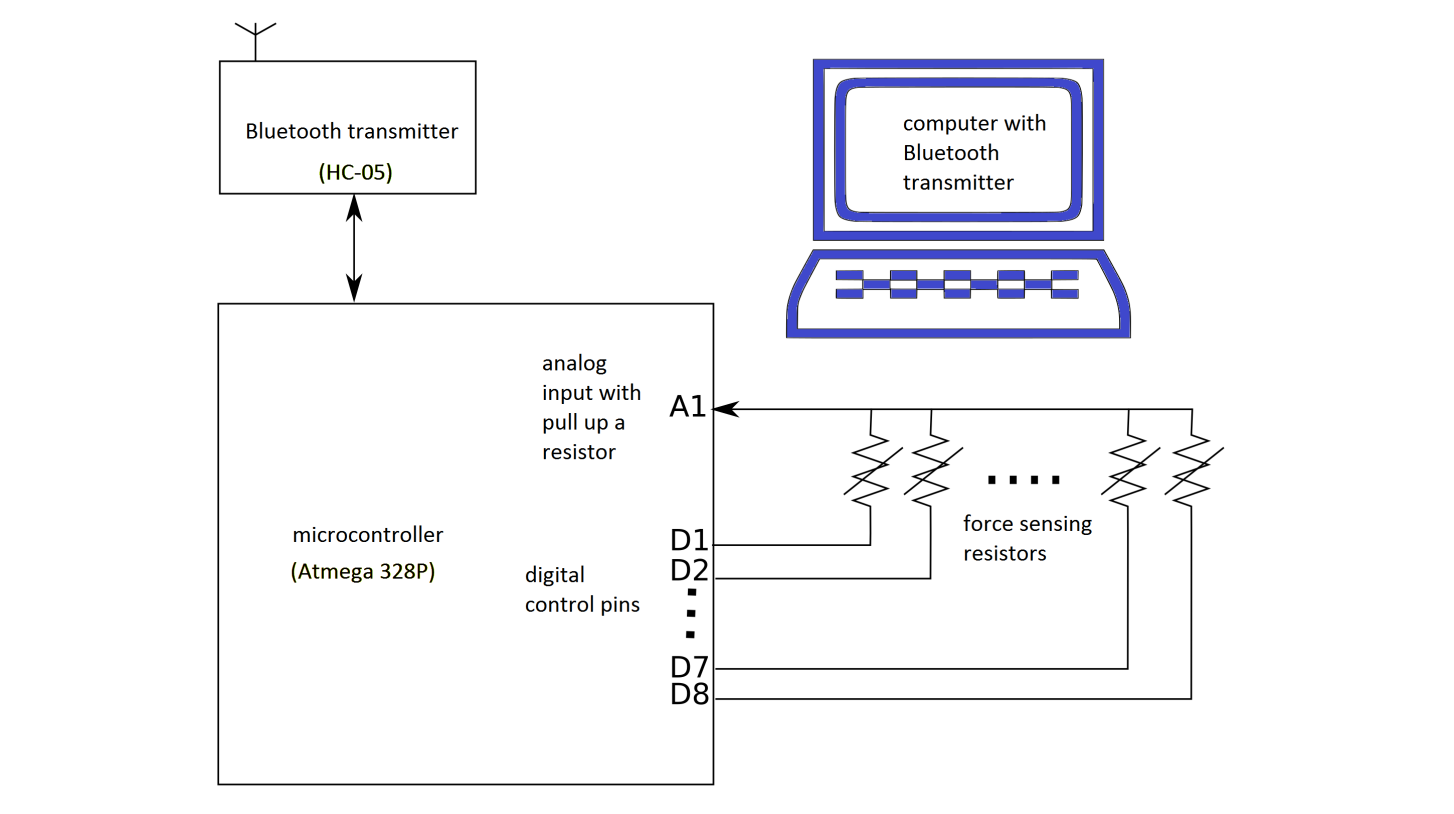
Schematic for FMG signal extraction and data transmission. The microcontroller sampled the signal from 8 FRS sensors, and the data were sent wirelessly to the computer through the Bluetooth transmitter module.

A single FSR sensor has 2 terminals: one terminal is connected to a common analog input pin of a microcontroller (Atmega 328p from Atmel) with an internal pull-up resistor (37.5 kΩ) equipped, and the other terminal is connected to a digital control pin as shown in Figure 2.

The analog input pin takes the reading of the signal and converts it into a 10-bit unsigned integer value. Since only 1 analog pin was used for sampling, the signal of each FSR was sampled sequentially. The order of sampling was determined by the digital control pin. When the selected FSR signal was ready to be sampled, the corresponding control pin was set to low, and the other control pins were set to be in high impedance states. At any single moment, only 1 control pin would be set to low and others would be changed to high impedance state in order to guarantee independent sampling. This configuration used a single analog pin with internal pull-up resistor in order to obtain the most simplified design under the constraints of the selected inexpensive microcontroller.

The sampled data were transmitted wirelessly to a personal computer using a generic Bluetooth module (HC-05). A custom-made application with real-time signal display was developed in LabVIEW on a personal computer for querying and storing the sample data. When sampling began, the application sent a command to the microcontroller to retrieve a set of FSR data at every 100 milliseconds (10 Hz) as proposed by Amft et al [40].

### Experimental Protocol

**Figure 3:**
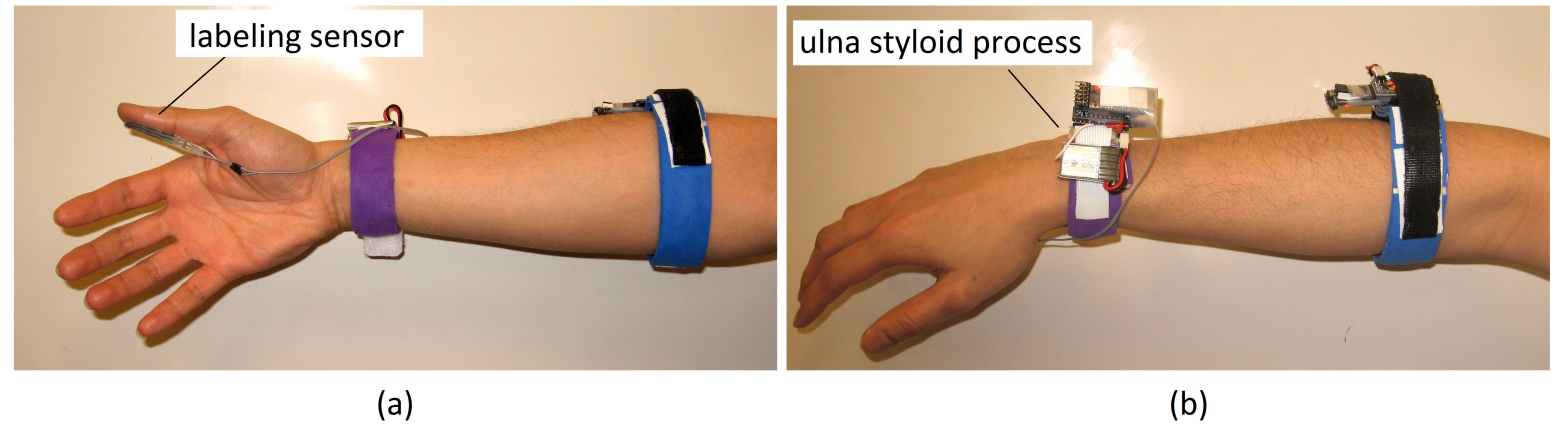
FSR straps placement: (a) forearm supinated view and (b) forearm pronated view.

An experimental protocol was designed to capture both wrist and forearm FMG signals simultaneously during a PAP exercise. Before the experiment, the forearm FMG strap was donned on the belly of the right forearm of a volunteer with the help of the research assistance. The wrist FMG strap was instead donned on the distal end of the forearm (next to the ulna styloid process, see Figure 3). In order to reduce signal inconsistency due to the placement of the strap for different volunteers, the first sensor near the tail end of the forearm strap (see Figure 1) was always placed on the bulk of the flexor carpi ulnaris, and the wrist counterpart was always placed near the ulna styloid process. However, it should be noted that the rest of the sensors were not positioned on specific muscles or tendons (the FSR were evenly distributed in the strap). This approach was intentionally followed to avoid personalization of the strap and provide a generic strap that could be used by a layperson at home. Finally, another FSR sensor (FSR 400 Interlink Electronics) was taped to the pulp of the thumb to obtain the true label for investigational purposes.

Once the straps were donned, the volunteer was asked to fully extend the fingers and then make a fist 3 times, while the research assistant monitored the raw signal through visual feedback from the display of the LabVIEW application. This hand action was shown to be able to generate a clear and visually distinguishable FMG pattern for healthy individuals [29,40,44], and therefore, the action was used to ensure the strap was able to register muscle-tendon movement activities. If this action did not generate a clear FMG pattern, the research assistant would readjust the tightness of the sensor. Also, the assistant would ensure the strap did not block blood circulation or cause discomfort to the volunteer through his or her oral feedback. On average, this calibration procedure took less than 3 minutes for each volunteer.

**Figure 4:**
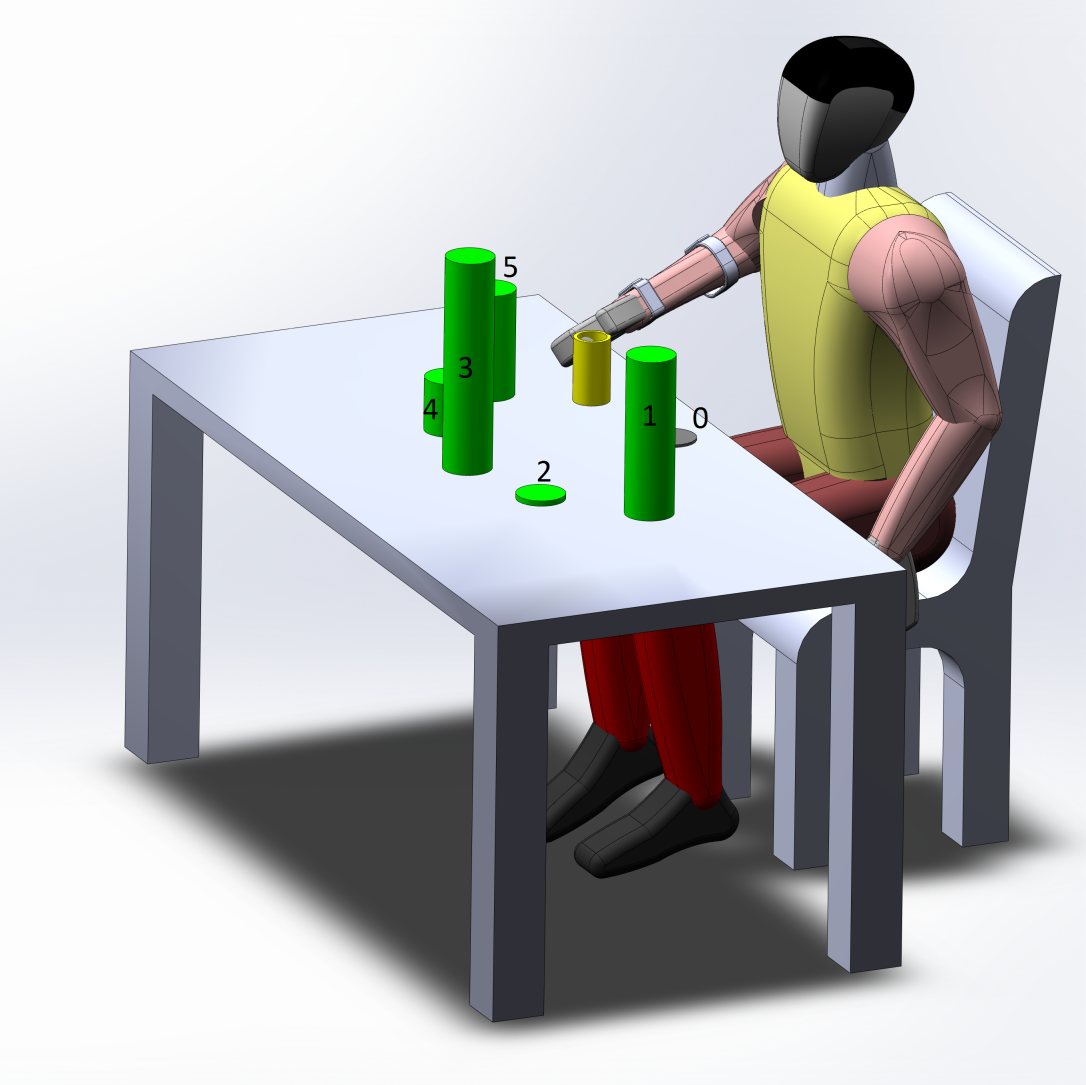
Experimental setup. The start location is shown in gray, the five target locations are shown in green, and the object for grasping is shown in yellow.

In the experiment, the volunteers sat in front of a table as illustrated in Figure 4. They were then asked to pick up and place a cylindrical object from and to 6 locations following different sequences. The object used in the experiment was a 12-cm high hollow cylinder with a radius of 3 cm. It weighed only 73 g so that the participants did not need to apply a large force to lift the object. The 6 locations included 1 start location (Location 0) and 5 other target locations (Locations 1-5). Locations 1-5 were placed around Location 0, at a distance of 40 cm. Using Location 0 as the reference, each of the 5 locations were 30 degrees apart from the adjacent one. The elevations of the 5 target locations were 30 cm, 1 cm, 40 cm, 10 cm, and 20cm from the table, respectively. Each location had a circular area with a 5-cm radius, such that the upper limb joints of the volunteer must be highly coordinated in order to successfully place the object on the target locations.

**Figure 5:**
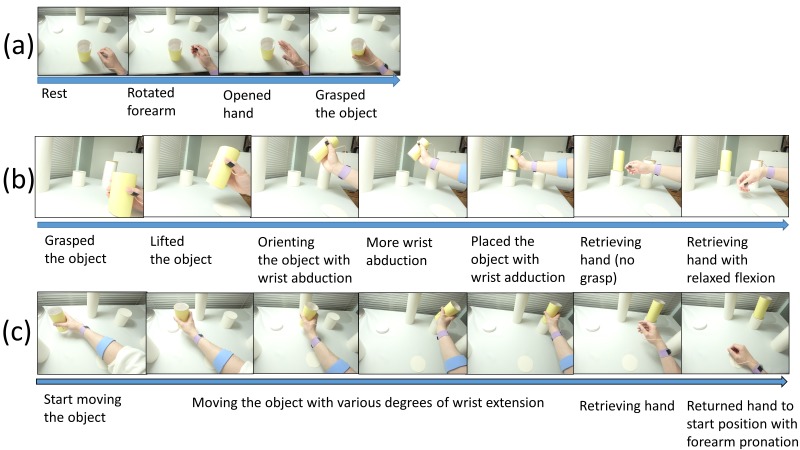
Examples of upper limb position during the PAP sequence: (a) grasping the object from start position; (b) transporting the object from start position to target location 4; and (c) transporting the object from target location 2 to 4.

In order to capture FMG signals in the presence of various arm movements, 3 PAP sequences were designed for the participants to perform. These PAP sequences required the coordination of the shoulder, the elbow, wrist, and hand. Therefore, the FMG patterns that were associated with some of the elbow flexion/extension, forearm pronation/supination, wrist flexion/extension/abduction/adduction, hand opening/closing, and the overall arm motion would be captured. Some examples of the captured movement during the PAP sequences are shown in Figure 5.

**Figure 6:**
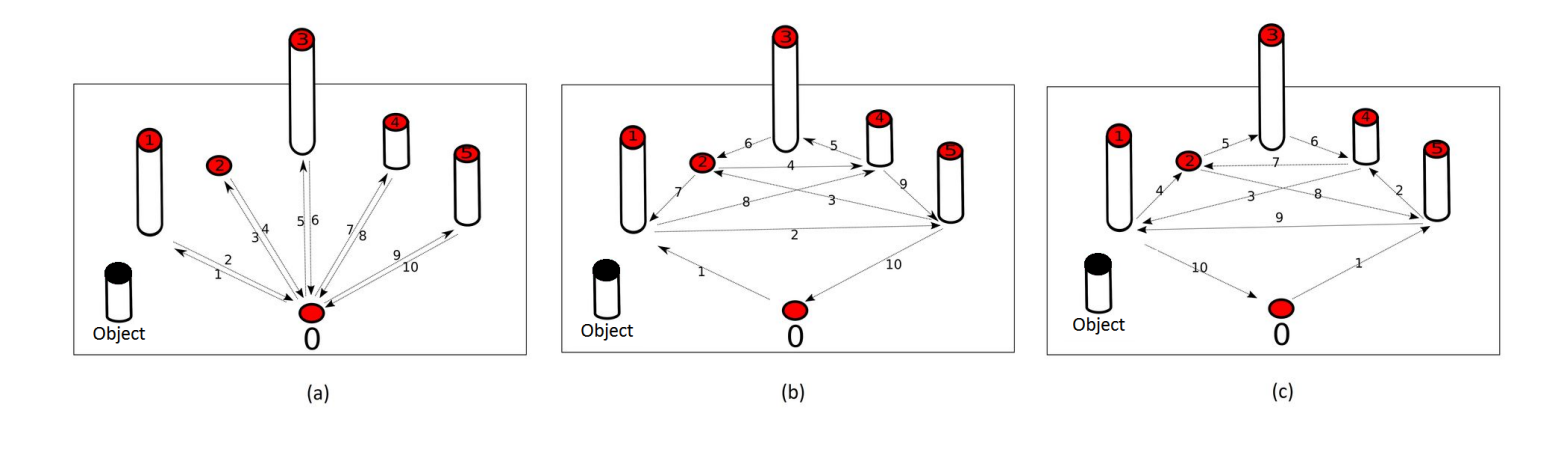
PAP action sequence. The red circles indicate the target positions, the arrows indicate the direction of the PAP actions, and the numerical labels indicate the orders of the steps in each sequence: (a) first PAP action sequence; (b) second PAP action sequence; and (c) third PAP action sequence.

In the first sequence, the participant was asked to pick up the object from the start location and place it onto the target locations at a pace comfortable for them. Then the participant retrieved their hand to the start location without the object. Next, the participant picked up the object from the current location, and returned it to the start location. Finally, the participant released the object completely before starting the next PAP action. In total, each participant performed 10 PAP actions for the first action sequence as shown in the left picture of Figure 6. In the second sequence, the participants performed an additional 10 PAP actions following the order shown in the middle of Figure 6. In the third sequence, the participants repeated the path of the second sequence but in a reversed order, as shown in the right of Figure 6. Each participant was asked to repeat the 3 sequences (30 distinct PAP actions in total) 5 times. With 3 sequences and 5 repetitions, a total of 150 PAP actions were recorded.

**Figure 7:**
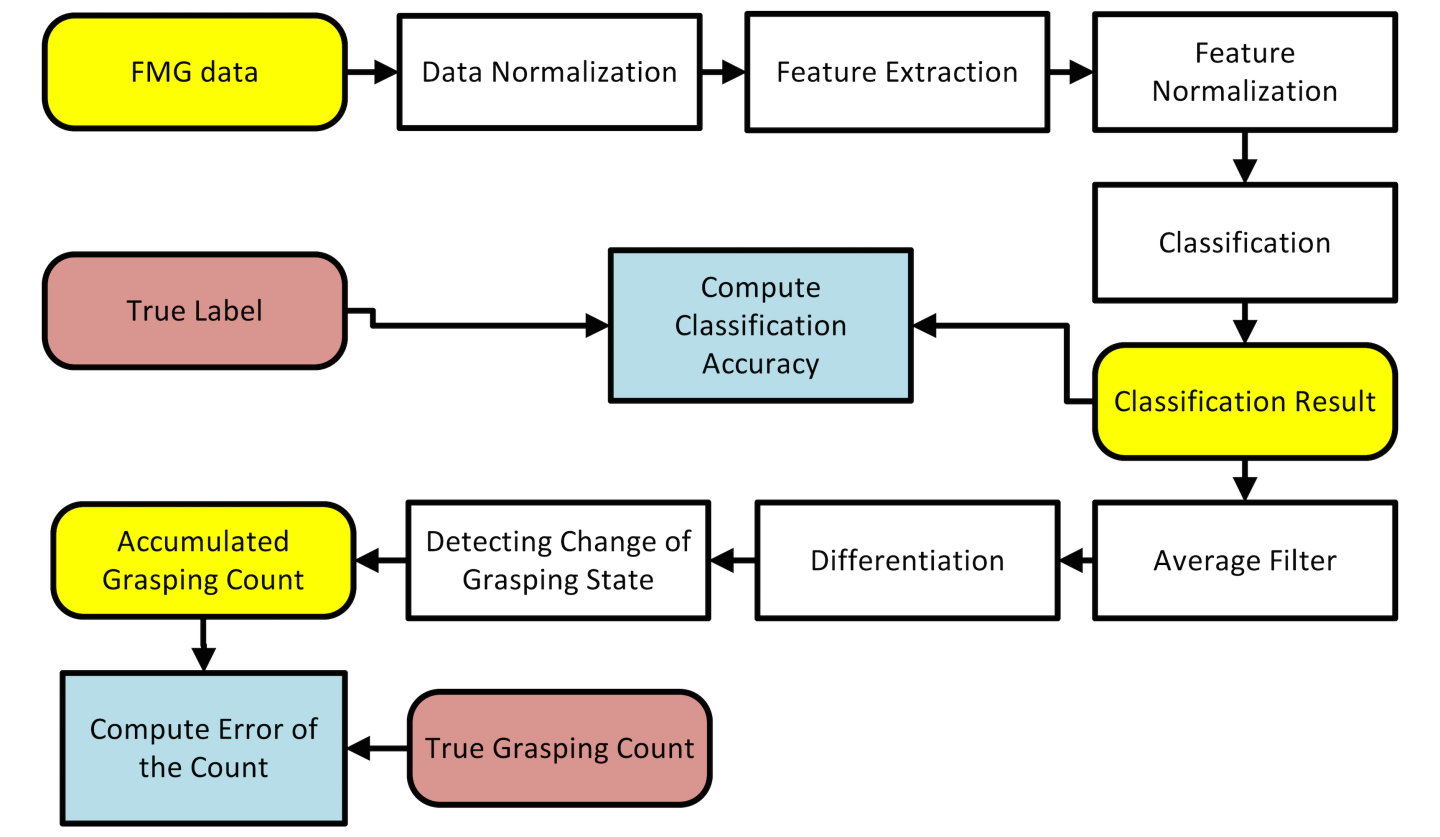
Data processing sequence.

### Participants

Ten healthy volunteers aged between 21 and 42 years participated in the experiment. Each participant signed an informed consent form (approved by the Office of Research Ethics, Simon Fraser University) before entering the study. Their wrist and forearm belly circumferences were recorded for performance analysis and are shown in Table 1.The average circumference of the wrist and forearm belly are 16.81 cm (SD 1.11) and 26.2 cm (SD 3.15), respectively.

**Table 1:** Participant statistics.

ID	Wrist circumference (cm)	Forearm belly circumference (cm)
1	16.5	28
2	19	27
3	17.5	27
4	16	27
5	17.6	28.5
6	15.5	27
7	16	17
8	18	26
9	16.5	26.5
10	15.5	28
Average	16.81	26.2
SD	1.11	3.15

### Data Processing

The data collected from each participant consisted of both wrist and forearm FMG positions. The 2 streams of data were processed through identical but independent treatments. The collected data were first divided into training and testing sets. The training set data consisted of the first 30 PAP actions, and the testing set consisted of the rest of the 120 PAP actions data sets. The training set was used for extracting relevant statistical information about the signals and for generating a classifier model. The testing set was used for examining the generalized performance of the classifier for detecting grasping. The overall data processing sequence is shown in Figure 7.

As shown in Figure 7, the raw FMG data of each channel was first centered by subtracting its mean and then normalized using its standard deviation. Both the mean and standard deviation parameters were obtained from the training set.

Next, feature extraction was performed. The raw FMG data were considered as primary feature of the signal. Three additional signal features, namely the root mean square (RMS), waveform length (WL), and window symmetry (WS), were extracted from the raw data with a 300 ms window and a step size of 100 ms.

**Figure 8:**
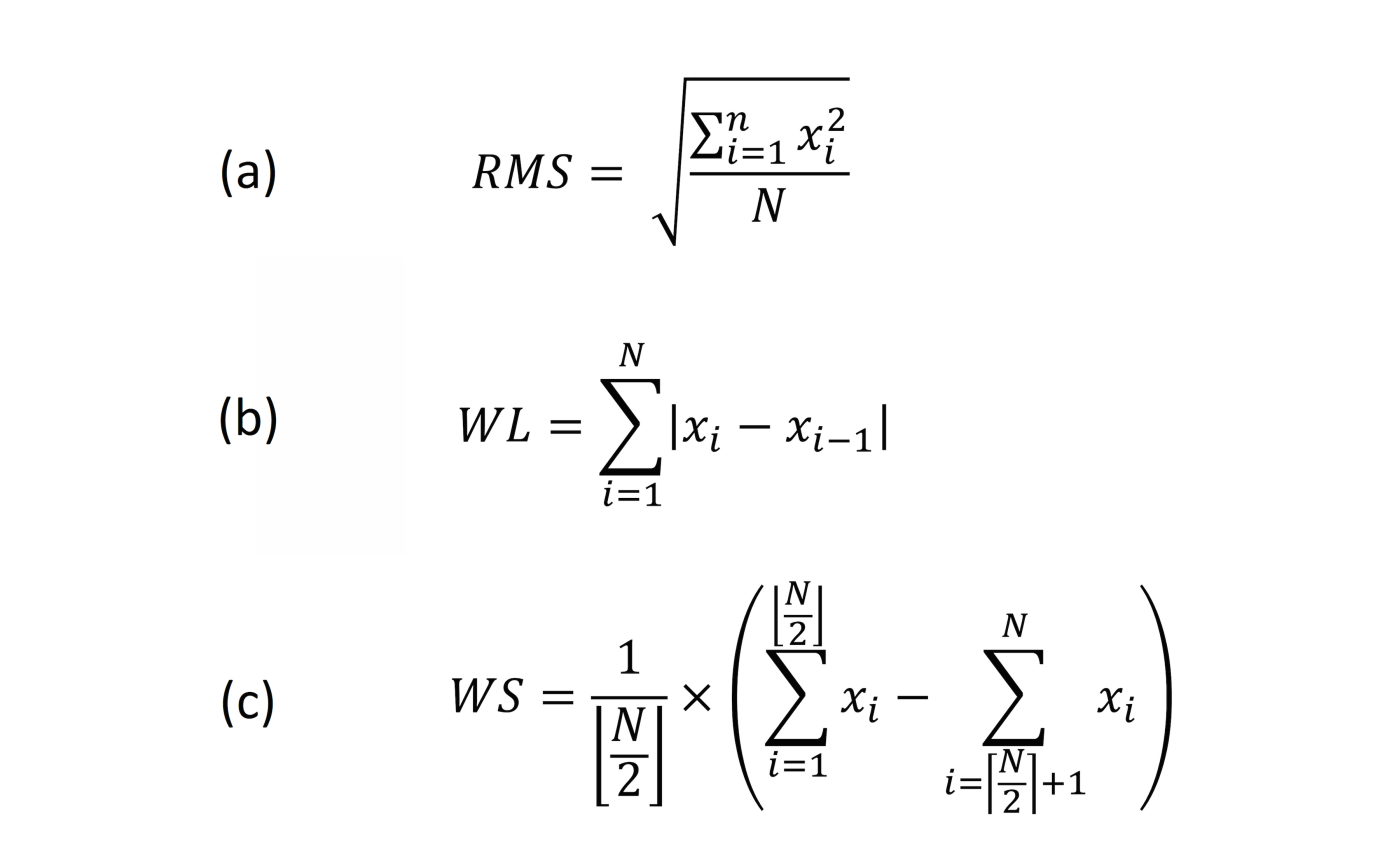
Equations for FMG feature extraction: (a) Root mean square, (b) Waveform length, and (c) Window symmetry.

RMS is the averaged signal magnitude of each window and its equation is shown in Figure 8. In the equation, *x*_i_ is the value of the *i*^th^ sample in the processing window and *N* is the window size, which in our case was 3.WL is the sum of the change of the input samples within the processing window, which provides speed-related information to the classifier. The formula for computing WL is shown in the middle of Figure 8.

WS is the difference between the average of the first *N* data points and the one of the last *N* data points, which can provide directional information of the change of the input samples. The formula for computing WS is shown in the bottom of Figure 8. A total of 4 features were extracted from each channel including the normalized raw input signal magnitude. Since there were 8 input channels for each of the wrist and forearm FMG straps, a total of 32 features were extracted, respectively. Each of the extracted features were once again centered and normalized based on their mean and standard deviation obtained from the training set before being classified using the supervised classification scheme.

Under the supervised classification scheme, the classifier needs to be trained using true label obtained from external source. In our case, the labeling signal was the one recorded by the FSR sensor placed on the thumb. This signal measured the amount of contact force between the object and the thumb. If the contact force was less than 2% of the maximum, then the corresponding FMG data was labeled as nongrasping (class 1); otherwise, the FMG data was labeled as grasping (class 2).

Among different supervised classifiers, linear discriminant analysis (LDA) classifier using Fisher discriminant criteria is one of the most widely used for analysis. LDA fits a multivariate normal density to each class with a pooled estimate of covariance. It is capable of revealing linear separability of the signal features. Additionally, LDA is computationally efficient and suitable to be implemented in a microcontroller [49]. Therefore, it was selected for use in this study.

The output of the LDA classifier was the predicted state of the hand. In order to count the number of grasping actions, the transition from nongrasping to grasping state needed to be identified. This transition could be detected by subtracting the current state output with the previous one. A positive result indicated a grasping action has occurred. However, the accuracy of such a counting method could be sensitive to any small glitches (misclassifications over a short period, eg, <1s) in the classification data output stream. Hence, an average filter was applied to smooth out the output stream of the classifier. The window size of the filter could affect the overall performance in terms of the counting accuracy and the delay. Therefore, the effect of different window sizes on the counting performance was examined (see Results section).

**Figure 9:**
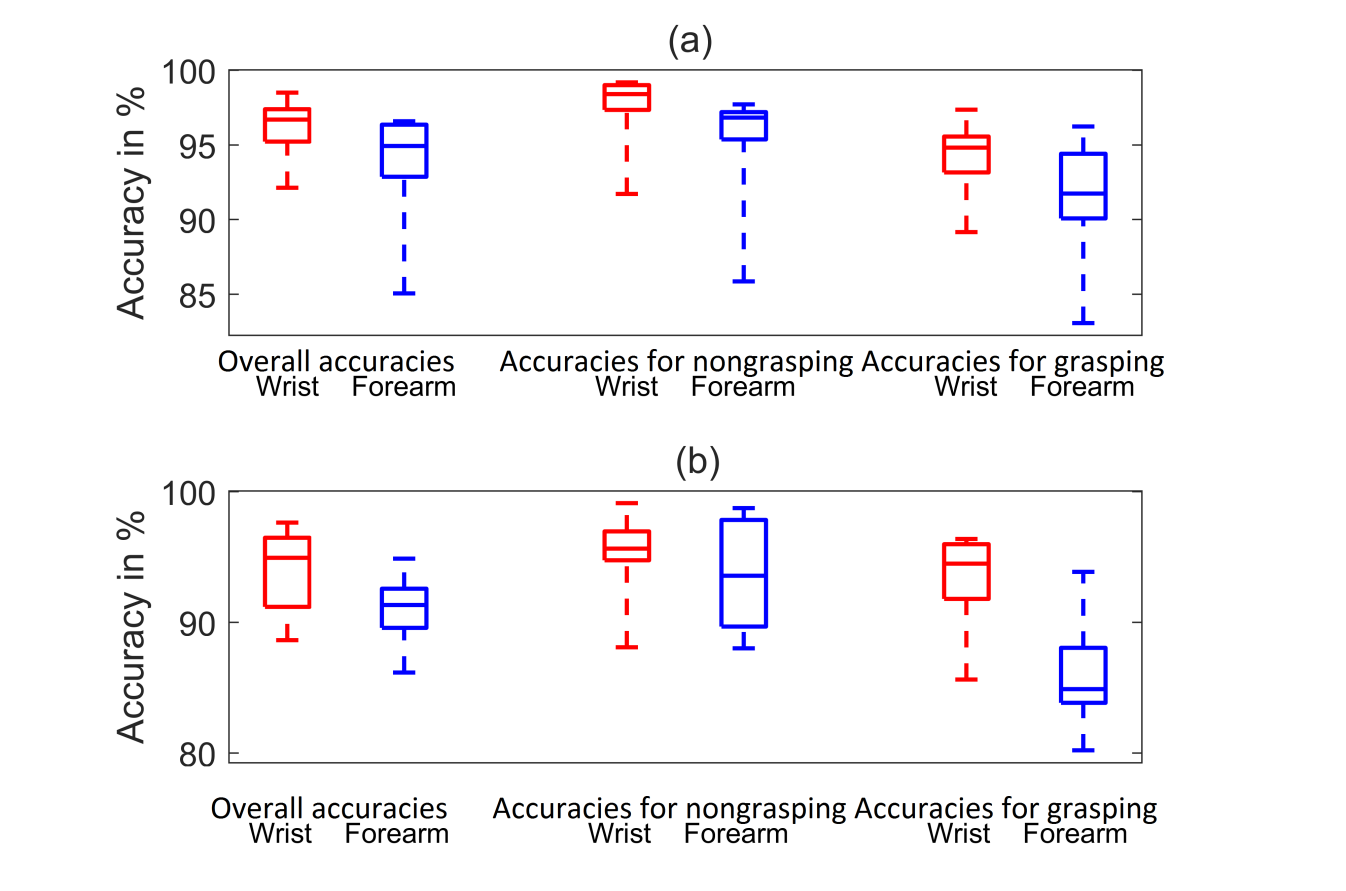
Boxplot for FMG classification accuracies of the 10 participants. The bottom and top of each box are the first and third quartiles of the data set, respectively. The band inside the box is the median. The ends of the dashed lines (whiskers) are the minimum and maximum of the data. The red and blue boxes indicate classification accuracies related to FMG collected from the wrist and forearm, respectively. (a) Accuracies computed using training set data. (b) Accuracies computed using testing set data.

**Figure 10:**
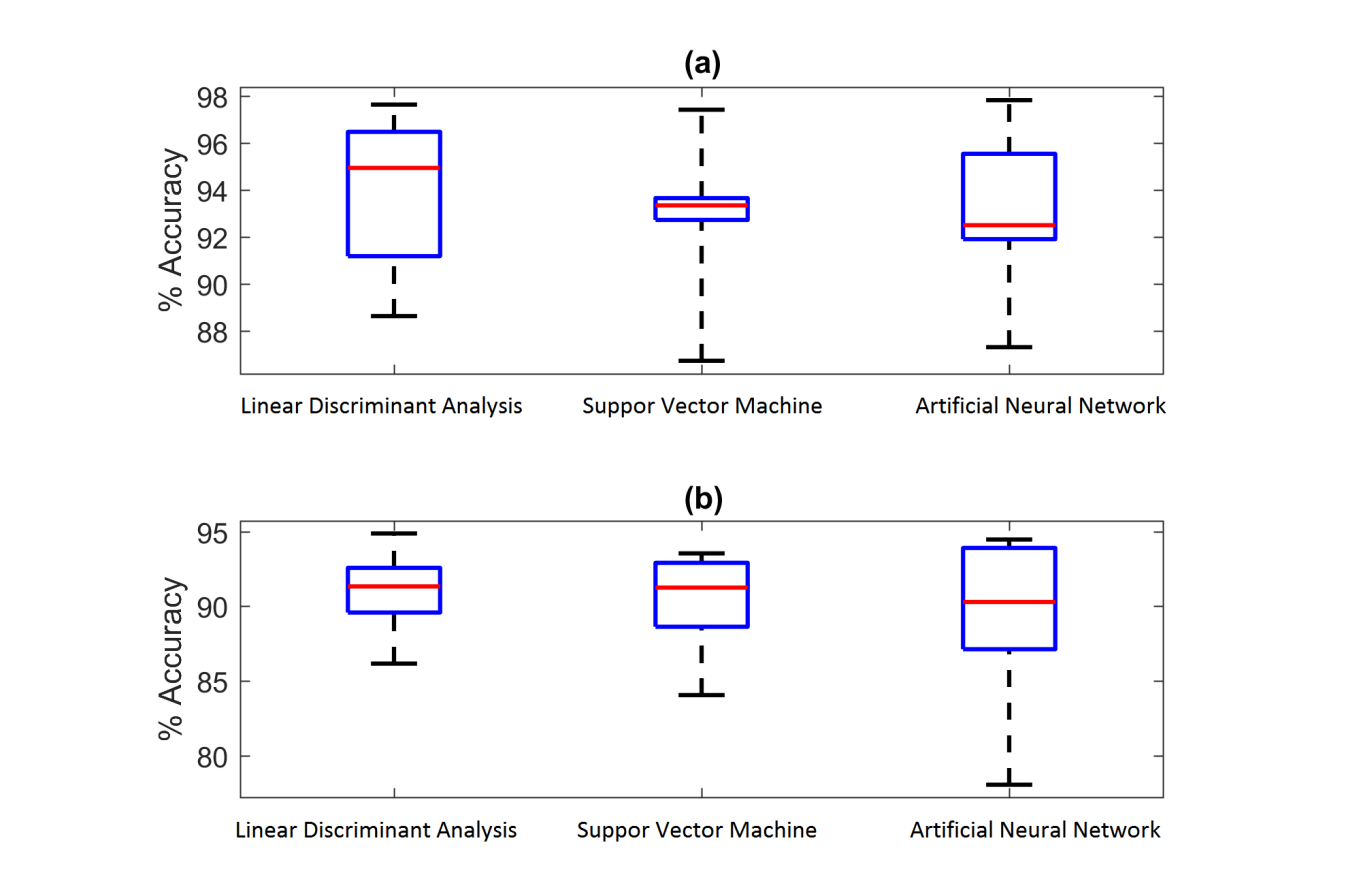
Classification result comparison: (a) accuracy comparison using wrist FMG and (b) accuracy comparison using forearm FMG.

**Figure 11:**
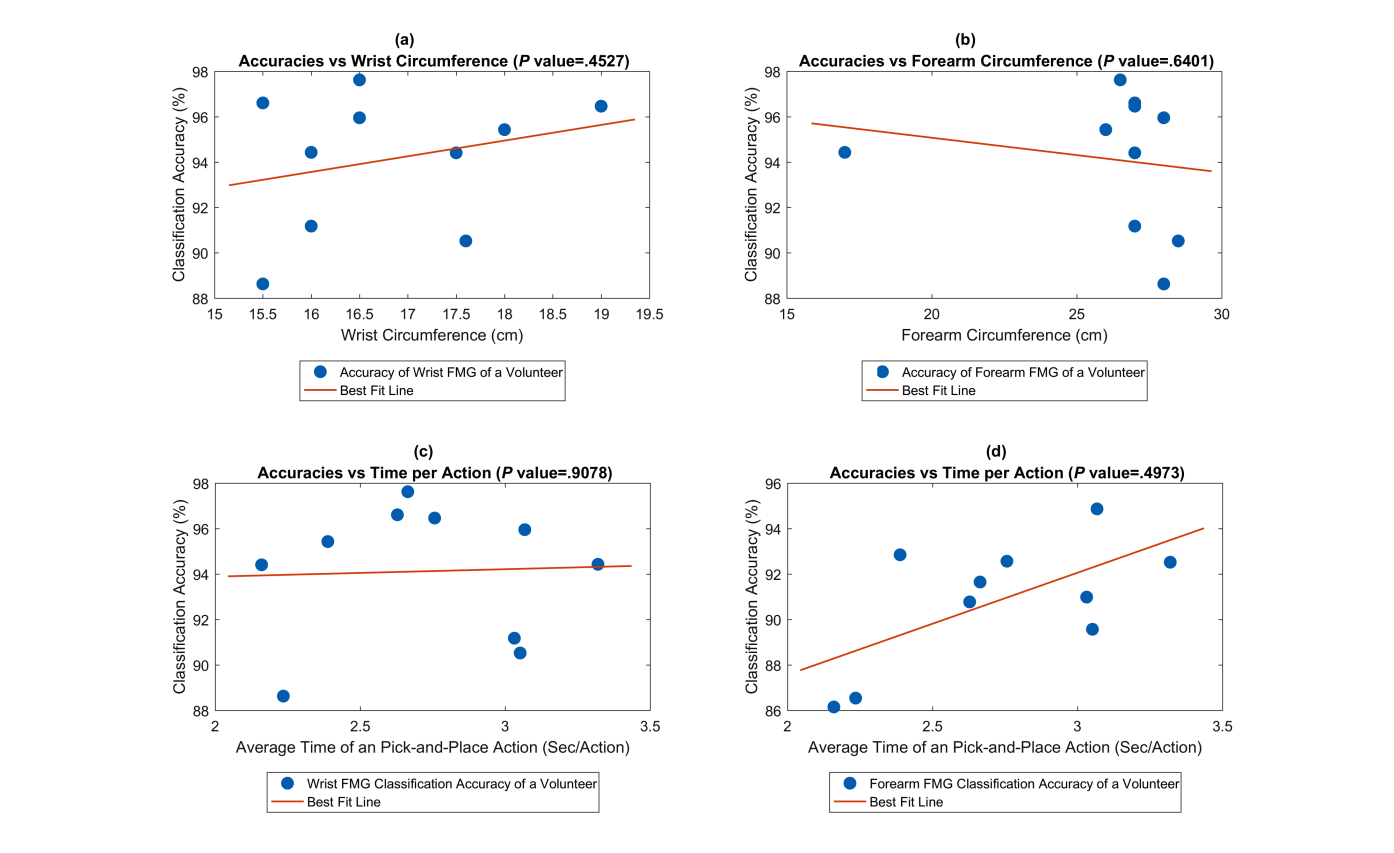
Regression plots for force-myography (FMG) classification accuracies of the 10 participants: (a) wrist FMG classification accuracies versus wrist circumference; (b) forearm FMG classification accuracies versus wrist circumference; (c) wrist FMG classification accuracies versus time per action; and (d) forearm FMG classification accuracies versus time per action.

**Figure 12:**
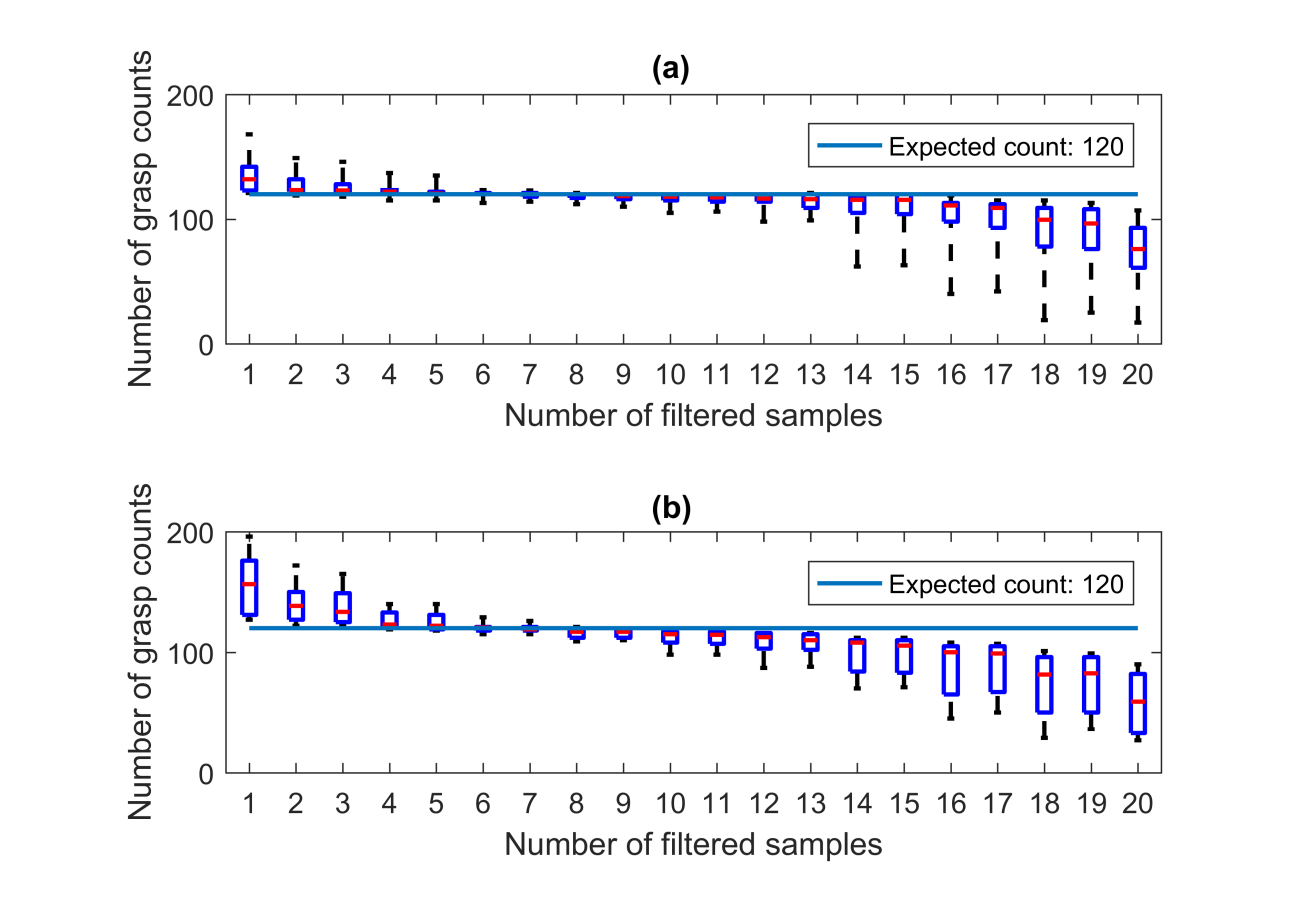
Boxplots for grasp counts versus number of filtered samples: (a) result generated using wrist FMG and (b) result generated using forearm FMG.

**Figure 13:**
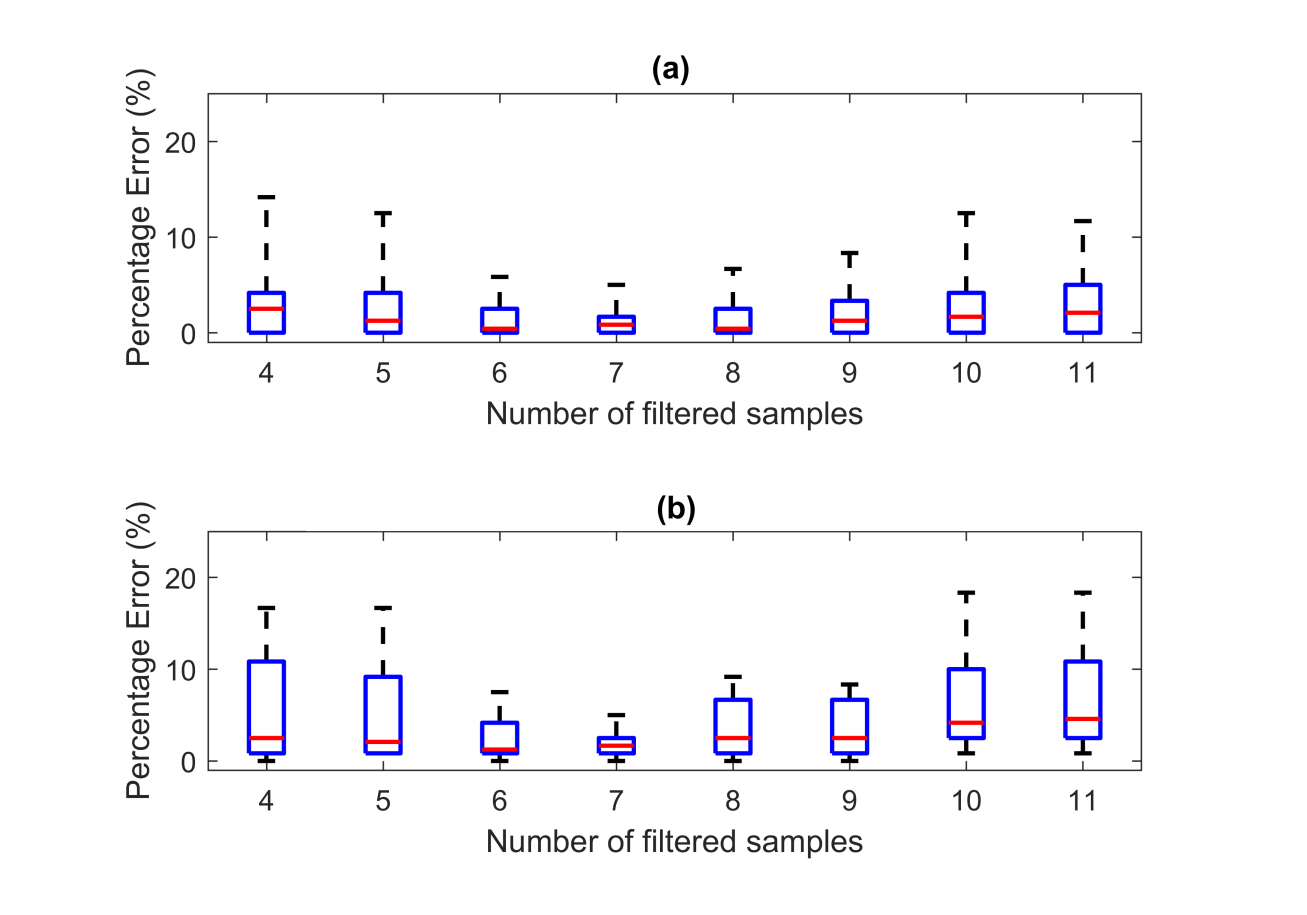
Boxplots for percentage error of grasp counts versus number of filtered samples: (a) result generated using wrist FMG and (b) result generated using forearm force-myography.

### Performance Evaluation

The overall performance of the proposed method was evaluated based on 2 metrics: the classification accuracies and percentage errors of the grasp count obtained from the test set.

The classification accuracy was calculated based on the sum of the correctly classified sample over the total number of samples. The difference of classification accuracies between wrist and forearm FMG using LDA was evaluated. Also, the performance of LDA was compared with other 2 popular classifiers, namely the Radial Basis Function kernel Support Vector Machine (RBF-SVM) and the 2-layer Artificial Neural Network (ANN). In addition, the correlations between the accuracies and action speeds, as well as the size of the wrist or forearm were assessed using a regression method.

The percentage error of the grasp count was based on the result of the absolute difference between the predicted and the expected counts over the expected one. The expected count for the test set was 120 in this study. The difference between the errors of the wrist and forearm FMG approaches was also assessed.

Paired sign test and Kruskal-Wallis test were used for examine the statistical significance of the obtained results. All the statistical analysis was performed using the same significance level (alpha) of .05.

## Results

### LDA Classification Results

The LDA classification accuracies of the 10 participants for nongrasping (class 1) and grasping (class 2) an object are shown in Figure 9. The combined accuracies of the 2 classes are shown in the first pair of results on the left of this figure (see “Overall accuracies”). Due to the fact that the results were not normally distributed, the median accuracy was used as the indicator for classification performance.

For the FMG recording from the wrist, the median training accuracy (see the top of Figure 9) was 97% and the corresponding interquartile range (IQR) was 2%. These high training accuracies suggest that FMG patterns recorded from the wrist are suitable to detect grasping and nongrasping during PAP actions. The median accuracy for the testing data set (see the lower plot of Figure 9) was 95% and the corresponding IQR was 5%. The high accuracies for both the training and testing data suggested that the training data was a good representation of the testing data set; no under- or overfitted phenomena was observed.

Similar results were obtained for FMG data recorded from the forearm: the median training accuracy was 95% and the corresponding IQR was 4%. The median testing accuracy was 91% and the corresponding IQR was 3%.

The difference between the medians of the wrist and forearm FMG testing accuracies was 4%. With a *P* value less than .001, the paired right-tailed sign test showed that the prediction using wrist FMG had a statistically significantly higher median accuracy than the one using forearm FMG.

The second pair of results in Figure 9 (see “Accuracies for nongrasping”) shows the prediction accuracies when the participants did not grasp the object. For FMG recorded from the wrist, the median testing accuracy was 96% and the corresponding IQR was 2% (see the lower plot of Figure 9). For FMG recorded from the forearm, the median testing accuracy was 94% and the corresponding IQR was 8%. With *P* value equals to .94, the paired right-tailed sign test did not show that the prediction accuracy of using wrist FMG was statistically different from the one using the forearm FMG for the nongrasping state.

The third pair of results in Figure 9 (see “Accuracy for grasping”) shows the prediction accuracies while the participants grasped the object. For FMG recorded from the wrist, the median testing accuracy was 95% and the corresponding IQR was 4%. For FMG recorded from the forearm, the median testing accuracy was 85% and the corresponding IQR was 4%. With a *P* value less than .001, the paired right-tailed sign test showed that FMG recorded from the wrist yielded a better prediction accuracy for grasping than FMG recorded from the forearm.

### Classification Result Comparison Between LDA and Other Classifiers

The performance of using LDA was compared with the ones of RBF-SVM and ANN. Standard model generation procedures for SVM and ANN, which are described in [50] and [51], were followed. For training the SVM model, a ten-fold cross-validation procedure was used to obtain best RBF parameters. For training the ANN model, a 2-layer network with 100 hidden nodes was trained based on a back-propagation algorithm. The obtained testing accuracies from all 3 classifiers are shown in Figure 10. For both wrist and forearm FMG classifications, no statistically significant difference among the results could be established using the Kruskal-Wallis test. The *P* values obtained from the test were .5 for wrist FMG classification and .9 for forearm FMG classification.

### Classification Results and Participants’ Physical and Performance Factors

In order to examine if the size of the participants’ limbs and action speeds influence the classification accuracies, 4 regression plots were generated and shown in Figure 11.

The first row of Figure 11 shows the regression plots of accuracies versus the wrist and the forearm circumference, respectively; the second row shows the regression plots of accuracies of the wrist and the forearm FMG classification versus the average time for the participant to complete a PAP action, respectively. With all *P* values larger than the specified significance level (alpha=.05), no statistically significant correlation could be established between accuracies and the 2 factors.

### Grasping Count Result

In order to assess the effect of the filtering window, the grasp counts of the participants were recomputed using different window sizes. Figure 12 shows the corresponding grasping count using box plot for the window sizes of 1-20 samples. The expected counts are shown as a solid line, which was 120 in this experiment.

Without averaging (sample size equals 1), the numbers of grasping were overestimated by a large margin for all the participants’ data. As the size of the averaging window increased, the counts were closer to the expected value in general. However, when the size continued to increase, the count became increasingly underestimated. On the basis of the result shown in Figure 12, medians of the counting error smaller than 5% were obtained when the number of filtered samples ranged between 4 and 11. The counting errors within such a range of filtered samples are shown in Figure 13.

As shown in Figure 13, the smallest maximum percentage errors were obtained by using 7 filtered samples for both wrist and forearm FMG counts (5% maximum percentage errors for both cases). Under such conditions, the median percentage error for wrist FMG was 1%, and the corresponding IQR was 2%. For the forearm FMG, the median percentage error was 2%, and the corresponding IQR was also 2%. A *P* value of .29 was obtained by using the paired 2-side sign test. Despite the wrist FMG having a smaller median, the statistical significant difference between the 2 FMG counts could not be established.

## Discussions

### Primary Findings

The LDA classifier was selected as the main classifier for this study, and its performance was as good as the more computational-intensive SVM and ANN classifiers. The LDA classification result (see Figure 9) shows that the wrist FMG band produced significantly higher classification accuracies than the forearm FMG band for detecting a grasping state, but no statistical difference was found for detecting a nongrasping state. This result could be associated to the fact that the grasping action occurred closer to the wrist than the forearm. During the object manipulation, movement of the thumb could be better registered by the wrist strap as the tendon and the nearby skin movements contributed to a more distinct FMG pattern. In addition, the FMG from the forearm also captured the pattern related to elbow movement [34], which was a confounding factor for grasp classification. Nevertheless, both FMG methods were capable of producing high classification accuracies (>85%) for all participants. These results confirmed that FMG was an effective method to detect hand grasping even with the presence of complex arm movements. The scope of this study focuses on the capability of FMG only, however, other wearable sensors such as an IMU should be considered along with FMG to further improve accuracy.

The regression method was used to test if the size of the participants’ limbs and action speeds influence the classification accuracies. The results of the test showed there were no statistically significant correlation between the accuracies and the 2 factors. These results suggested the performance of the FMG method was independent of the limb size and action speed in this study. However, since the participants in this study were healthy individuals, the variations of their physical status and action performance were expected to be insignificant. For future investigation, grasping at various speeds by individuals with different limb sizes should be considered.

The grasping counts were extracted from the filtered output of the LDA classifiers. As shown in Figure 12, the size of the filtering window largely influenced the accuracies of the counts. Suitable window sizes (medians of the counting error smaller than 5%) were identified to be from 4 to 11 samples, and such window sizes could add 300 ms to 1000 ms of delays to the system. Combined with the delay introduced by the feature extraction process (300 ms), a grasping could be registered by the system after at least 600 ms from the start of the action. While such a delay might be problematic for some real-time human machine interfaces [52-54], it is not considered to be of concern for the targeted activity monitor application, as the user’s instant action does not depend on the feedback [55-57]. Under the optimal settings, which was based on the smallest maximum percentage error, a median percentage error of 1% with IQR of 2% for the grasping count using the wrist FMG was obtained. Compared with the results obtained using forearm FMG, no statistically significant difference was established using the paired sign test. These results show great potential for both FMG approaches.

This study investigated the capability of using FMG to predict and count grasping actions with healthy individuals. Therefore, the knowledge obtained from this study can be directly applied to the applications in which healthy individuals are the wearers of the FMG bands. An example of such an application could be for monitoring the repetitive hand activity level of a worker during load transfer tasks. However, for rehabilitation applications, as the targeted population will be the people with a weak arm, such as individuals recovering from stroke, further studies are needed to examine the transferability of the result of this study. For example, in poststroke rehabilitation, the targeted wearers are often seniors with limited mobility and range of motion. Their muscles are normally much weaker than the ones of healthy individuals, and may even have significantly deteriorated if the individuals are chronic stroke patients. These characteristics posed questions on whether the proposed FMG method could be used with such a population. Currently, there is lack of in-depth studies that examine the FMG pattern characteristic of stroke patients or people with a weak arm. However, the pilot investigation of Yungher and Craelius showed that the grasping force could be regressed from the forearm of stroke patients (n=4) with mild to moderate spasticity [36]. This study indicated some useful FMG information could still be extracted as long as the patients had some range of motion on the limb. For object manipulation task, this type of patient tends to produce larger grip force, but with less control when compared with the healthy counterpart [58,59]. In such a scenario, distinct FMG patterns associated with some movements are still expected to be captured; however, the consistency of the patterns is likely to be less. The inconsistency due to muscle fatigue may also become more prominent, which might require modification of algorithms to adjust the training parameter of the classifier (eg, normalization parameters of the FMG signals) in order to compensate for the change. In addition, the FMG pattern can be very different among patients due to the different degree of impairment. Because of these conditions, the classifier model may need to be very user- and task-specific in order to tailor for the needs and obtain high prediction accuracy. In order to examine the transferability of the proposed FMG approach for rehabilitation, testing on the stroke population or individuals with weak arm should be the next logical step.

### Conclusions

The possibility of detecting and counting grasping in the presence of arm movements (PAP exercise) was explored using wrist and forearm FMG strap prototypes. The 2 main performance parameters that were considered were classification accuracy for detecting grasping and percentage error for counting grasping. A high median grasping prediction accuracy was obtained from 10 subjects (95% and 91% for FMG recorded from the wrist and forearm, respectively). A low median grasping count error was also found (1% and 2% for wrist and forearm, respectively). These results provide evidence that FMG-based straps could be used to monitor grasping activities during functional arm movements in a controlled environment. This work poses the foundation for future studies investigating the applicability of using FMG to detect grasping in activities of the daily living first with healthy participants and then with individuals with a weak arm (eg, seniors, individuals with a hemiparetic arm resulting from stroke).

## Abbreviations

ADL: activity of the daily living
ANN: artificial neural network
FMG: force myography
FSRs: force-sensing resistors
IMU: inertial measurement unit
IQR: interquartile range
LDA: linear discriminant analysis
RBF-SVM: Radial Basis Function kernel Support Vector Machine
RMS: root mean square
sEMG: surface electromyography
WL: waveform length
WS: window symmetry
